# The innate immune brakes of the lung

**DOI:** 10.3389/fimmu.2023.1111298

**Published:** 2023-01-27

**Authors:** Catherine Sabatel, Fabrice Bureau

**Affiliations:** ^1^ Laboratory of Cellular and Molecular Immunology, GIGA-Research, University of Liège, Liège, Belgium; ^2^ Faculty of Veterinary Medicine, University of Liège, Liège, Belgium

**Keywords:** lung, homeostasis, immunoregulation, airway allergy, innate immunity

## Abstract

Respiratory mucosal surfaces are continuously exposed to not only innocuous non-self antigens but also pathogen-associated molecular patterns (PAMPs) originating from environmental or symbiotic microbes. According to either “self/non-self” or “danger” models, this should systematically result in homeostasis breakdown and the development of immune responses directed to inhaled harmless antigens, such as T helper type (Th)2-mediated asthmatic reactions, which is fortunately not the case in most people. This discrepancy implies the existence, in the lung, of regulatory mechanisms that tightly control immune homeostasis. Although such mechanisms have been poorly investigated in comparison to the ones that trigger immune responses, a better understanding of them could be useful in the development of new therapeutic strategies against lung diseases (e.g., asthma). Here, we review current knowledge on innate immune cells that prevent the development of aberrant immune responses in the lung, thereby contributing to mucosal homeostasis.

## Introduction

The “self/non-self model”, which has dominated Immunology since the 1950s, states that an immune response is triggered against any foreign (i.e., non-self) antigen encountered by the immune system, whereas no immune response is triggered against the organism’s own constituent (i.e., self) (1, 2). More recently, Polly Matzinger proposed a rival theory, called the “danger theory”, which claims that immune responses are triggered only when antigens are accompanied by “danger signals” or “alarmins” derived from injured or stressed cells (damage-associated molecular patterns; DAMPs) or pathogens (pathogen-associated molecular patterns; PAMPs) (3, 4).

**Figure 1:**
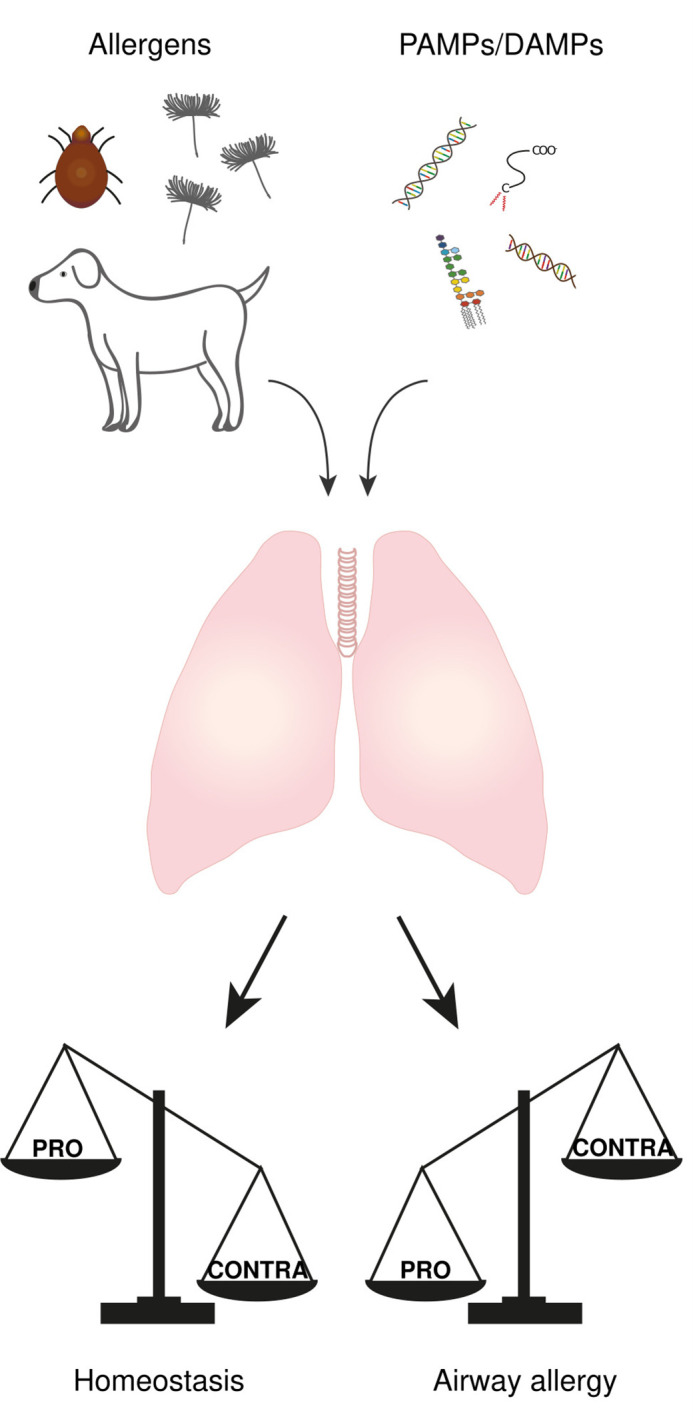
The balance between “PRO” and “CONTRA” mechanisms determine the immunological outcome of an antigen encounter in the lung. The lung is continuously exposed to both airborne antigens and immunostimulatory molecules called PAMPs and DAMPs. The development of an immune response or not is the result of an imbalance between some “PRO” (i.e., mechanisms that drive the immune response) and “CONTRA” mechanisms (i.e., mechanisms that prevent the immune response), where “PRO” mechanisms prevail in airway allergic people while “CONTRA” mechanisms predominate in most people leading to homeostasis.

The respiratory tract is continuously exposed to both innocuous airborne antigens (i.e., non-self antigens) and immunostimulatory molecules such as endotoxins (lipopolysaccharides; LPS) released by Gram-negative bacteria (i.e., danger signal). According to either “self/non-self” or “danger” models, this should systematically result in the development of immune responses toward these inhaled harmless antigens, such as T helper type (Th)2-mediated allergic reactions. However, only a small fraction of people develops airway allergy (5, 6), suggesting that mechanisms exist that tightly control lung homeostasis and prevent aberrant immune response. One might even argue that the development of an immune response in the lung only occurs when “PRO” mechanisms (i.e., mechanisms that drive the immune response) overtake “CONTRA” mechanisms (i.e., mechanisms that prevent the immune response). In this view, it is likely that “CONTRA” mechanisms prevail in most people exposed to both harmless antigens and immunostimulatory molecules, leading to homeostasis, whereas “PRO” mechanisms predominate in patients who develop allergic asthma (**Figure 1**).

These immune brakes are far from being totally understood as most research focuses on elucidating and identifying the cells and pathways driving the development of immune response. Nevertheless, it is generally accepted that regulatory T cells (Tregs) are the most important cells in maintaining immune tolerance in the lung. However, if Treg development was the normal outcome of harmless antigen encounter, mediastinal lymph nodes (MLNs) draining the airways would be the place of incessant Treg proliferation, which is highly improbable in view of the small size of MLNs in uninfected and unsensitized mice and the low percentage of Treg in these lymph nodes (7). It is therefore reasonable to think that Treg form a second (adaptive) line of defense against the development of aberrant immune response, and that other innate cells, located in the lung rather than in the MLNs, must constitute the first (innate) line preventing the development of exaggerated immune responses. The myeloid-derived suppressor cells (MDSCs) constitute another well-known population of immunosuppressive cells (8). This heterogenous population, defined by their T cell immunosuppressive functions, appears in cancer and in pathologic conditions associated with chronic inflammation or stress (9). However, their absence at steady state in healthy individuals makes them unlikely to sustain lung homeostasis.

In this article, we review current knowledge on cells endowed with immunoregulatory properties in the lung, mainly focusing on innate immune cells. MDSCs will not be discussed as they have been well-documented since their initial description in the 1970’s (10) and reviewed several times recently (8, 11).

## Macrophages and monocytes

Macrophages are the first immune sentinels of the airways. Due to their ability to induce Th1 immune response (12), they have been considered as a target of choice to alleviate Th2-mediated allergic responses. Several studies aimed at demonstrating that antigen-stimulated macrophages are able to mediate suppression of allergic airway inflammation in mice showed that the transfer of antigen (Ag)-pulsed macrophages to sensitized mice led to a decrease of airway eosinophilia and hyperresponsiveness to methacholine (13–17). The Ag-specific IgE and Th2 cytokine production by T cells upon Ag stimulation were also reduced (13–17). This immunosuppressive response appeared to be antigen-specific and long lasting (15) and was not associated with an increased Th1 profile, arguing against Th1-mediated counter-regulation (16). However, many of these studies were conducted with non-pulmonary macrophages [i.e. peritoneal (13–15) and immortalized splenic macrophages (16)] meaning that these studies were more focused on therapeutic strategies than shedding light on cells sustaining lung homeostasis. Regarding the mechanisms displayed by these macrophages, the G protein coupled receptor GPR101 was shown to be a regulator of peritoneal macrophages phenotype and function, limiting the propagation of inflammation and expediting its resolution (18).

The steady-state lung contains two different macrophage subsets, the alveolar macrophages (AMs) and the interstitial macrophages (IMs), residing in the alveolar space and in the lung interstitium respectively. Besides their different localization, these cells can be easily discriminated based on their phenotypic profile in lung without inflammation. In addition to typical macrophage markers (e.g. F4/80, MertK, CD64), at the steady state, mouse AMs express CD11c and SiglecF while IMs express CD11b and CX3CR1 (19, 20). Regarding their function, it is generally accepted that AMs provides primary defense *via* clearance and phagocytosis of incoming pathogens (20–25) while IMs exert immunoregulatory activities (20, 21, 25–27). However, AMs immunosuppressive functions have also been reported. Both subsets have been described in the human lung. Many similarities between mouse and human macrophages have been reported based on marker expression, location, function and even developmental pathway, especially for AMs, the most studied lung macrophages (19, 20, 28, 29).

### Alveolar macrophages

The ability of AMs to prevent inappropriate immune responses has been inferred from depletion experiments prior to (30, 31) and after antigen sensitization (17, 31–33). Intratracheal instillation of clodronate-filled liposomes enables the specific depletion of phagocytic cells localized in the airway lumen, i.e. AMs in vast majority. The depletion is therefore transient and non-specific to AMs *per se*. More recently, AMs were also depleted using CD169-DTR mice (34). However, caution should be taken when interpreting the results obtained using these transgenic mice to target AMs as IMs also express CD169, even though less than AMs (35). *In vivo* elimination of AMs using clodronate-filled liposomes led to overt inflammatory reactions in sensitized mice to model antigens, such as trinitrophenyl-keyhole limpet hemocyanin (TNP-KLH) (30), ovalbumin (OVA) (17, 32, 33) or house dust mites (HDM) (31, 33). Increased IgE levels and development of mononuclear cell infiltrates in the lung were also observed following AMs depletion in OVA-sensitized rats (36). AMs exerted their immunosuppressive properties toward HDM no matter whether their depletion occurs prior the sensitization or challenge phase (31). Clodronate treatment during the sensitization phase resulted in a reduction of HDM-induced IL-27 concomitant with exacerbation of Th2 pathology, suggesting a role for IL-27 in regulating Th2 responses at mucosal surfaces (31). Surprisingly, AMs depletion alleviated the trimellitic anhydride (TMA)-induced drop in lung function parameters observed in TMA-sensitized rats (37). The levels of serum IgE were also decreased (37). In contrast, TMA-induced tissue damage and inflammation were augmented following AMs elimination (37). Indeed, AMs seemed to suppress non-specific inflammation caused by TMA conjugated to endogenous protein (TMA-BSA) challenge (38). In line with this study, transfer of naïve AMs to OVA-sensitized AMs-depleted mice resulted in decreased airway hyperreactivity and eosinophil counts in the bronchoalveolar lavage (BAL) fluid whereas no improvement was observed upon transfer of sensitized AMs (32). The same phenomenon was observed in rats (39), suggesting that allergen sensitization modulates AMs function. AMs phagocytosis was although diminished in sensitized AMs (39) underscoring the importance of AMs status for their control of the pulmonary response in a suppressive way.

**Figure 2:**
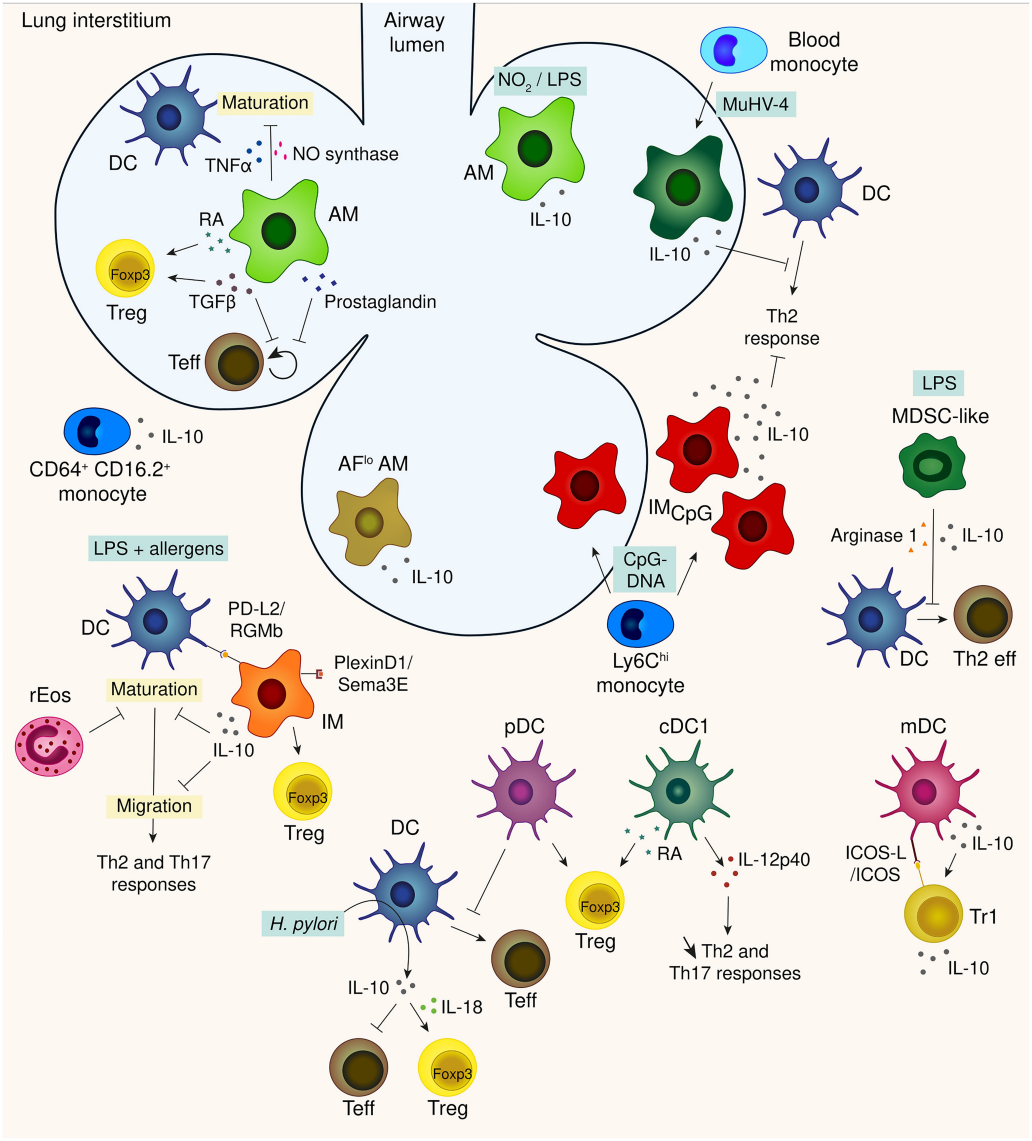
A synthetic view of the innate immune brakes of the lung.

Looking at the mechanisms, *in vitro* co-culture of rat AMs with antigen presenting cells (APCs) across a semipermeable membrane revealed an inhibition of APC maturation, amplified by TNF-α and abrogated *via* blockade of the nitric oxide synthase pathway (40). When mixed with T cells, AMs appear to allow T-cell activation and expression of T-cell effector function, while selectively inhibiting T-cell proliferation (41) (**Figure 2**). This suppression involves a unique form of T-cell anergy, associated with inhibition of IL-2 receptor signal transduction (42). The induction of unresponsiveness was reversed upon removal of AMs from the T cell (42) or upon granulocyte-macrophage colony-stimulating factor (GM-CSF) treatment (43) in rodents and by the addition of CD28 costimulation or IL-2 in human (44). Rodent and human AMs also differ in the mechanisms employed to achieve this inhibition: rodent AMs appear to utilize reactive nitrogen intermediates, while this does not appear to be the case for human AMs (41). Nevertheless they both release prostaglandin and TGFβ (45, 46) suggesting that pulmonary macrophages use multiple mechanisms for locally suppressing lymphocyte activation. More recently, apoptotic cell uptake by lung AMs was shown to suppress HDM-driven allergic asthma while dampening AMs capacity to make inflammatory cytokine, increasing their responsiveness to adenosine (whose receptor limit allergic inflammation upon agonist treatment) and their retinoic acid (RA) production (47). In line with this study, mouse AMs were also shown to induce regulatory T cells (Tregs) *in vitro* through the release of RA and TGFβ (48, 49) (**Figure 2**) even though IMs appear to be more potent in inducing the expression of the forkhead box P3 transcription factor, Foxp3, the master regulator of Tregs, in naïve T cells (50). These Treg-inducing AMs were nevertheless able to promote airway tolerance as their transfer into sensitized mouse airways prevented the development of asthmatic lung inflammation upon subsequent challenge with Ag (49). Several mediators from the lung microenvironment such as TGFβ production, SIRPα and CD200R stimulation and low doses of nutrients are also able to drive AMs toward tolerogenic function, preventing potentially detrimental lung inflammation (51).

NO_2_ exposure induces the infiltration of an AM subpopulation in rodent BAL fluid that may exert anti-inflammatory functions by the production of high amounts of the immunosuppressive cytokine IL-10 (52). In the same way, delivery of low-dose LPS in mice to prime the lung was shown to augment AMs production of IL-10 in the BAL fluid and enhance resolution of lung inflammation induced by a lethal dose of LPS or by Pseudomonas bacterial pneumonia (53) (**Figure 2**). On the other hand, a study revealed that mouse AMs do not produce IL-10 upon LPS stimulation *in vitro* (54). These results are in accordance with others, showing that IL-10 production by mouse AMs is quiet low in comparison to mouse IMs, using IL-10-β-lactamase reporter (ITIB) mice (35, 55) and *in vitro* culture with LPS-containing OVA stimulation (56). Caution should be taken when considering all BAL fluid cells as AMs since the presence of IMs was shown in mouse BAL fluid upon stimulation with CpG-DNA (35). Moreover, inflammatory stimuli induce phenotypical changes among AM and IM populations making their discrimination more complex. Recently, two subpopulations of macrophages have been described in the human BAL fluid based on their degree of autofluorescence and their ability to secrete IL-10 (57) suggesting that a fraction of human IMs could be present in the airway lumen.

### Interstitial macrophages

Besides their localization in the lung interstitium, IMs have been defined as regulatory macrophages. Indeed, since the first description of their regulatory function in 2009 (56), many studies confirmed that IMs produce IL-10 in the steady state (35, 58–60) and can in this way contribute to lung homeostasis.

Upon LPS-containing OVA exposure, *ex vivo* cultured IMs were found to impair the ability of co-cultured bone marrow-derived dendritic cells (BMDCs) to maturate, migrate to the draining lymph node and induce features of Th2-mediated airway allergy once reinjected into the trachea of recipient mice (56). This effect was mediated through IL-10 production by IMs since *Il10^-/-^* IMs failed to do so (56) (**Figure 2**). *In vivo*, mouse IMs were shown to be localized in close vicinity of lung dendritic cells (DCs), which are endowed with the ability to trigger allergen-specific Th2 responses (56), and IMs-depleted mice developed airway allergy following exposure to low doses of allergens and LPS (56). The immunosuppressive potential of IMs is nevertheless surpassed by the high dose of the allergens and LPS, given that 100% of WT mice develop asthma upon exposure to high amounts of HDM extracts, a phenomenon that requires Toll-like receptor 4 (TLR4) activation by HDM-borne LPS (61). IMs also regulate Th17-mediated inflammatory response since their transfer to HDM-exposed *Il10^-/-^* mice reduce the Th17-related neutrophilic inflammation (59).

IL-10 production by IMs is increased upon inflammatory stimuli such as HDM, LPS, CpG-DNA, Flagellin and FSL-1, a synthetic lipoprotein derived from *Mycoplasms salivarium* (35, 56, 58, 59). CpG-DNA is by far the most potent stimulator of IL-10 in mouse IMs and it also induces a dramatic expansion of IL-10-producing IMs (35). These CpG-induced IMs were able, by producing IL-10, to confer protection against allergic inflammation even when mice were sensitized and challenged with high doses of HDM (35) (**Figure 2**). Intranasal delivery of mesenchymal stem cell-derived exosomes was also able to substantially expand lung IL-10-producing IMs and thus contributed to protection against allergic asthma in mice (62).

Signaling pathways that promote IL-10 expression in IMs have been little studied but the constitutive production of IL-10 in IMs was shown to be mediated through activation of the TLR4/myeloid differentiation factor 88 (MyD88) pathway in a microbiota-independent manner (59). In allergenic contexts, MyD88-dependent upregulation of the transcription factor Hypoxia-inducible factor 1-alpha (Hif1α) boosts the expression of IL-10 by lung IMs (58). Recently, semaphoring 3E/plexinD1 signaling in IMs was shown to be a critical pathway for their immunoregulatory activity as IMs genetic deficiency in plexinD1 impaired IL-10 production leading to airway allergy in mice exposed to HDM (63) (**Figure 2**).

While IL-10 production is the most studied immunoregulatory mechanism used by IMs, it is not the only one. The interaction of repulsive guidance molecule b (RGMb), expressed on IMs, with programmed death ligand 2 (PD-L2), expressed on DCs, appear to be essential for respiratory tolerance. Indeed, blockade of the RGMb-PD-L2 interaction markedly impaired the development of respiratory tolerance in a mouse model of tolerance to OVA (64). Lung IMs were also shown to induce the proliferation and differentiation of Treg cells (50) (**Figure 2**).

Functional studies on IMs have been mainly conducted in mice, probably due the difficulties faced to isolate macrophages from healthy human tissue. However their existence have been described in human and non-human primates (60, 65). Human IMs produce IL-10 more potently than human AMs, mainly upon LPS stimulation but also at steady state (60) and would be functionally impaired in asthmatic patients (66). Recently, a population of macrophages with properties similar to IMs was described in the human BAL fluid (57). These cells, identified as autofluorescent^low^ (AF^low^) AMs in comparison to classical AF^high^ AMs, expressed a unique transcriptional signature associated with specific immunoregulatory functions, including the ability to secrete IL-10 (57) (**Figure 2**). Such signature, along with their small size is reminiscent of what is described for IMs in the murine lung indicating that some humans “IMs” might be present in the airway lumen. This would facilitate investigations on human IMs.

Recent studies have begun to reveal heterogeneity among IMs compartment (50, 55, 67) with at least two main populations with different phenotypes and localizations in mice (50, 55). Mouse CD206^+^ Lyve1^hi^ MHCII^lo^ IMs are mainly located in the bronchial interstitium (55) and associated to blood vessels (50) while mouse CD206^-^ Lyve1^lo^ MHCII^hi^ IMs are mainly located in the alveolar interstitium (55), and associated to nerve bundles (50). Regarding their function, CD206^+^ IMs produce more IL-10 than CD206^-^ IMs (50, 55). On another hand, CD206^-^ IMs were shown to induce Treg more potently than CD206^+^ IMs, in accordance with their high expression of MHCII (50). A study showed that MHCII^hi^ IMs can be further divided on two subsets based on CD11c expression (67). However, differences in function between these two subsets has not been investigated. A population of mouse CD169^+^ IMs, named nerve and airway-associated macrophage and described as a new subset of IMs, was reported recently (68). However, their phenotypic analysis reveals that these cells largely overlap with CD206^-^ IMs identified previously. In humans, two subsets transcriptionally similar to murine subsets were also described (50). However, unlike mouse IMs, MHCII (HLA-DR) cannot be used to identify human IM subsets as this marker is expressed at higher levels in CD206^+^ Lyve1^+^ IMs (50).

### Monocytes

Two main populations of monocytes have been described in the mouse lung: the Ly6C^hi^ GR-1^hi^ classical monocytes and the Ly6C^lo^ GR-1^lo^ patrolling monocytes (69) with their human counterparts consisting of CD14^+^ CD16^-^ and CD14^lo^ CD16^+^ monocytes respectively (70). However, lung monocytes are mainly located in the blood vessels associated to the lung (35). Only a fraction of Ly6C^hi^ monocytes, called Ly6C^hi^ lung monocytes (35), and a discrete population of CD64^+^ CD16.2^+^ N4RA1-dependent (patrolling monocytes key transcription factor) monocytes (55) are truly located in the mouse lung tissue. Monocytes can extravasate into the lung tissue where they can differentiate into tissue macrophage or DC or recirculate to lymph nodes without any differentiation (71). Most of the monocytes and monocyte-derived cells harbor proinflammatory properties and contribute to the development of immune response. Nevertheless, some regulatory functions have been reported.

First of all, a fraction of lung resident CD64^+^ CD16.2^+^ monocytes were shown to express IL-10 (55), suggesting regulatory properties. Regarding Ly6C^hi^ lung monocytes, CpG-DNA exposure induced their differentiation into hypersuppressive CpG-induced IMs (35). Splenic monocytes were also recruited to the lung to constitute the pool of CpG-induced IMs (35). Infection with the murid herpesvirus 4 (MuHV-4) was shown to inhibit the development of HDM-induced experimental asthma by modulating lung innate immune cells (72). This immunosuppressive effect was attributed to monocytes that replenished resident AMs upon MuHV-4 infection (72). These monocyte-derived AMs displayed regulatory properties, including IL-10 production, and blocked the ability of DCs to trigger a HDM-specific response by Th2 cells in mice (72) (**Figure 2**). MuHV-4-imprinted monocytes are also able to recruit CD4 T cells to the airways and trigger immunosuppressive signaling pathways through the PD-L1/PD-1 axis, thereby dampening the deleterious activation of cytotoxic CD4 T cells (73). Monocytes can also act as suppressor cells that promote Treg development (74). Indeed, adoptive transfer of GR-1^+^ monocytes in tumor-bearing mice revealed the differentiation of such monocytes into tolerogenic DCs that produce IL-10 and potently induce Treg response and expansion (74). During gut infection, monocytes can acquire regulatory properties in the bone marrow thanks to a priming by natural killer (NK) cells (75). This process could potentially occur in other mucosa like the lung. A population of GR-1^+^ cells was observed in ozone-exposed mice (76). These cells were diminished in the absence of CX3CR1 and appeared to protect the host from the biological response to ozone (76). Indeed, CX3CR1-null mice exhibited enhanced responses to ozone, including increased airway hyperresponsiveness, exacerbated neutrophil influx, accumulation of 8-isoprostanes and protein carbonyls and increased expression of cytokines (76). This population was identified by the authors as a novel macrophage subset, distinct from AMs. Despite their expression of F4/80, a major macrophage marker (77), these cells do not look like any population of lung macrophage already described and highly express GR-1 (76), a monocytic marker. As mentioned earlier, inflammation makes discrimination between cell population harder due to phenotypic changes and overlapping marker expressions, so it is possible that these cells are stuck at an intermediate state between classical monocytes and macrophages.

Human CD14^+^ monocytes are potent activators of TGFβ, *via* expression of the integrin αvβ8 and matrix metalloproteinase 14, which dampens their production of TNFα in response to LPS (78). In the healthy human intestine, a mucosa which, like the lung, have to deal with foreign compounds, integrin αvβ8 is highly expressed on mature tissue macrophages, with these cells and their integrin expression being significantly reduced in active inflammatory bowel disease (78). This suggests a key role of integrin αvβ8-mediated TGFβ activation in the regulation of inflammatory responses and mucosal homeostasis by monocytes and macrophages.

## Dendritic cells

The idea that DCs are able to induce tolerance *in vivo* originated from experiments on DCs that are not fully mature (79). These immature DCs were shown to inhibit T cell proliferation (80, 81) and to induce Treg cells (81) through IL-10 production (82). DCs were also treated with IL-10 (83) or engineered to express IL-10 (84, 85) in an attempt to develop therapeutic strategies. Since then, some physiological counterpart of these *ex vivo*-derived DCs were identified as some resident lung DCs were shown to exert immunoregulatory properties.

At the steady state, the lung comprises two main populations of DCs, plasmacytoid DCs (pDCs) and conventional DCs (cDCs), also called myeloid DCs (mDCs), the latter subdivided into two functionally distinct subsets, type 1 and type 2 cDCs. Despite different surface markers expression between human and mouse cDC subsets, the transcription factors interferon regulatory factor 8 (IRF-8) and basic leucine zipper ATF-like transcription factor 3 (Batf3) drive the development of cDC1 while IRF4 drives the development and terminal differentiation of cDC2 in both species (86–89).

In addition to their production of type I interferon upon viral infection, lung pDCs were shown to induce tolerance. Indeed, in a mouse model of tolerance, an increase of pDCs in the lung draining lymph node was reported (90). Moreover, pDC depletion during inhalation of normally inert Ag led to IgE sensitization, airway eosinophilia, gobelet cell hyperplasia and Th2 cytokine production while adoptive transfer of pDCs before sensitization or challenge prevented such features in mice (91, 92). On a functional level, mouse pDCs did not induce T cell division in the lung but suppressed the generation of effector T cells induced by cDCs (90, 91). They were also shown to induce *in vitro* the differentiation of Treg cells capable of suppressing Ag-specific T cell proliferation (91, 93) (**Figure 2**). Lung pDCs exhibited these tolerogenic properties irrespective of their maturation state since the efficiency of CpG-matured pDCs and immature pDCs were the same, through programmed death (PD)-1/PD ligand (PDL) 1 interactions but not through ICOS ligand, IDO and IFNα unlike splenic pDCs (92, 94). Although human pDCs were discovered long before their mouse counterparts, their identification in human lung failed for a long time (88, 95, 96). However, human pDCs isolated from blood or lymphoid tissues efficiently promote the generation of CD4^+^ CD25^+^ Foxp3^+^ IL-10-producing Treg cells (97–99), suggesting that human lung pDCs might play a role in the maintenance of immunological tolerance.

Lung cDCs are also endowed with regulatory properties. A study even reported that mice lacking CD11c^hi^ lung DCs, but containing pDCs, failed tolerization with inhaled Ag and could not support Foxp3 induction *in vivo* in naïve CD4^+^ T cells (100). The different conditions and mouse models used in these studies are probably responsible for this discrepancy.

Likewise, mDCs from mice exposed to OVA were reported to transiently produce IL-10 (101). The adoptive transfer of these DCs isolated from OVA-exposed mice prevented the recipient mice from the development of airway inflammation (101). The protection was mediated through IL-10 production by DCs since the adoptive transfer of *IL-10^-/-^* DCs failed to protect from Th2-mediated inflammation (101). In line with that, a study revealed that IL-10 production by DCs is diminished in atopic children (102). In mice, IL-10-producing DCs exhibited a mature profile and stimulated the development of CD4^+^ T regulatory 1-like cells that also produced high amount of IL-10 (101) through a pathway involving ICOS-ICOS ligand (103) (**Figure 2**). Despite their expression of CD11c, no further phenotypic characterization has been done on these IL-10 producing DCs that would allow these cells to be more clearly identified.

Digging a little deeper into identifying a cDC subset endowed with immunoregulatory properties, several studies have shown that *Batf3^-/-^* mice, which are devoid of cDC1, challenged with Ag failed to develop tolerance and developed exacerbated Th2 and Th17 immune responses and exacerbated airway inflammation (100, 104, 105). Mechanistically, Batf3 absence does not affect induction of Treg or IL-10 production by lung CD4^+^ T cells following Ag challenge but impaired IL-12p40 production (104). IL-12 treatment reverts exacerbated allergic airway inflammation in *Batf3^-/-^* challenged mice, restraining Th2 and Th17 responses without triggering Th1 immunity (104), suggesting a protective role for lung cDCs 1 in allergic airway inflammation through the production of IL-12. Lung cDCs 1 were also shown to be able to induce Foxp3 in naive CD4^+^ T cells. They upregulated retinaldehyde dehydrogenase 2 (aldh1a2) (100), which is a key enzyme involved in the production of a cofactor for TGF-β to induce Foxp3 expression. RA-producing DCs were accordingly identify in the lung (106) (**Figure 2**). Thus, lung cDCs 1 would induce Treg differentiation through RA production as demonstrated for gut musocal cDCs 1 (107, 108). Regarding cDCs 2, their expression of C5aR1 was shown to promote tolerance towards aeroallergen such as OVA and HDM through downregulation of CD40 (109). Different stimuli can also modulate DC function orienting them toward immunoregulatory profile. Indeed, mouse and human cDCs were shown to produce IL-10 upon *Helicobacter pylori* exposure, subsequently protecting from allergen-induced asthma in mouse models (105). *H. pylori* was also reported to inhibit LPS-induced maturation of DCs and reprogram DCs toward a tolerance-promoting phenotype (110). These reprogramed DCs failed to induce T cell effector functions and instead induced expression of FoxP3 in naïve T cells through IL-18 production (110) (**Figure 2**). Lipid mediators such as peroxisome proliferator-activated receptor (PPAR)-γ agonists and prostaglandin D_2_ were also shown to inhibit DC migration to the MLN and reduce the T-cell response in the MLN in OVA sensitization mouse models (111, 112).

## Granulocytes

### Eosinophils

Accumulating evidence indicates that, besides their pro-inflammatory roles in Th2 responses associated with helminth infections or allergic diseases, eosinophils also regulate homeostatic processes at steady state and exhibit protective role under certain conditions (113, 114).

At steady state, the mouse lung contains resident eosinophils (rEos) which display unique morphological and phenotypical features that unambiguously distinguish them from the inflammatory eosinophils (iEos) that are recruited to the lung during HDM-induced allergic airway inflammation (115). CD101 is the main characteristic that enable to distinguish rEos from iEos: rEos do not express CD101 while iEos are CD101^+^ (115). These rEos were shown to inhibit the maturation, and therefore the pro-Th2 function, of allergen-loaded DCs and correspondingly, mice lacking lung rEos showed an increase in Th2 cell response to inhaled allergens (115) (**Figure 2**). In human, the parenchymal rEos identified in non-asthmatic lungs were phenotypically distinct from the iEos isolated from the sputa of eosinophilic asthmatic patient, suggesting that the findings in mice are relevant to humans (115). Mouse lung eosinophils play also a crucial role in lung allograft acceptance. While associated with rejection of other solid organs, local nitric oxide (NO) generation is critical for lung allograft acceptance (116). Eosinophils were shown to be the dominant inducible NO synthase (iNOS)-expressing cells in the lung allograft and their depletion reduced NO levels to that of recipient mice and led to allograft rejection (117). NO production by eosinophils depends on stimulation by IFN-γ and TNF-α since neutralization of such mediators in graft recipients abrogates eosinophil suppressive capacity (117). The iNOS^+^ lung eosinophils were phenotypically similar to the previously described lung rEos, indicating that rEos may display several immunoregulatory functions.

In guinea pigs, ozone exposure induced eosinophil hematopoiesis which limit ozone-induced airway hyperreactivity since depletion of these newly recruited eosinophils worsened airway hyperreactivity (118). This ozone-induced hematopoiesis of beneficial eosinophils was blocked by TNF-α antagonist or by prior allergen sensitization, suggesting that atopic individuals might have worsened airway hyperreactivity following ozone exposure or delayed resolution of symptoms because of a lack of bone marrow response (119).

### Neutrophils and mast cells

Like for eosinophils, emerging evidences point out regulatory functions for neutrophils (120). Indeed, it was shown that neutrophils can decrease DCs function (121–123), protect host from LPS-induced lethal inflammation (124) and produce anti-inflammatory molecules such as IL-10 and act as T-cell suppressors in different contexts (125–128). However, these functions have been mainly attributed to circulating neutrophils and immunoregulatory properties of lung neutrophils have been poorly investigated. So far, a study revealed that mycobacteria-infected DCs attract neutrophils that produce IL-10 and specifically shut down the otherwise exuberant Th17 response in the mouse lung (127).

Besides their well-known roles in allergy and innate immunity, mast cells have also the potential to turn immune responses off (129, 130). Although an immunosuppressive role has not been uncover yet in the lung, several protective properties have been reported in the skin where mast cells are important to suppress UVB-induced contact hypersensitivity (131), limit leukocyte infiltration in contact dermatitis (132), impair the development of Ag-specific T cell response following *Anopheles* mosquitoes bites (133) and induce an optimal tolerance to skin allograft through Foxp3^+^ Treg cells (134).

## MDSC-like cells

The hygiene hypothesis postulates that living in a microbe-rich environment reduces the risk of developing asthma (135–138). Several studies have uncovered mechanisms that may underlie this phenomenon, such as exposure to CpG-DNA inducing high amount of IL-10 producing-IMs (35) or early exposure to MuHV-4 inducing the replacement of AMs by regulatory monocytes (72). A study also showed that in mice, continual exposure to LPS induced the generation of a suppressive myeloid cell type that express CD11b, GR-1 at intermediate levels and F4/80, distinguishing it from neutrophils, macrophages and DCs but resembling myeloid-derived suppressor cells (139). LPS promoted the development of MDSC-like cells, that were both phenotypically and morphologically similar to those described in the tumor environment, in a TLR4/MyD88-dependent manner (139, 140). These cells did not traffic to the lung-draining lymph node but blunted the ability of the lung DCs to upregulate GATA-3 or to promote STAT5 activation in primed Th2 cells, both transcription factors having critical roles in Th2 effector function (139). This effect was reversed by anti-IL-10 or inhibition of arginase 1 (139) (**Figure 2**).

## Lymphoid cells

In addition to Treg cells, other lymphoid cells have regulatory potential. Indeed, it was shown that regulatory type of B cells (Breg) play a critical role in the development of T cell tolerance to aeroallergens (141, 142) and that their deficiency increases allergic airway inflammation in mice (143). Depletion of mouse CD8 T cells before prior immunization lead to increased Th2 responses (144) and these cells seem to play important role in the negative regulation of IgE production and airway responsiveness (145). Moreover, γδ T cells deficient mice are naturally hyperresponsive upon airway challenge (146). However, the regulatory properties of these lymphoid cells are subject to debate since they have generally been described under certain conditions uniquely as these cells can paradoxically also promote Th2 responses (144, 147).

A subset of regulatory innate lymphoid cells (ILCreg) that produce IL-10 have been described in mouse and human intestine (148). These cells play regulatory role in intestinal homeostasis akin to Treg cells (148). In the lung, RA was shown to convert ILC type 2 (ILC2, i.e. IL-5 and IL-13-producing ILC) to IL-10-producing ILCreg (149).

## Epithelial cells

Epithelial cells are key cells in the maintenance of pulmonary homeostasis. Besides their physical role in immune regulation, epithelial cells can communicate with innate immune cells to mount adapted immune responses or to dampen them. Indeed, their production of cytokines such as IL-33 and thymic stromal lymphopoietin (TSLP) are responsible for the activation of different innate immune cells (i.e. DCs and ILC2) that promotes the development of immune responses (150–152). However, in link with hygiene hypothesis, farm dust was reported to induce the ubiquitin-modifying enzyme A20 in epithelial cells, modifying their communication with DCs and thus protecting from allergy development (153). Moreover, epithelial cells were shown to intercommunicate with AM to reduce endotoxin-induced lung inflammation (154) and to control inflammatory signaling through signal transducer and activator of transcription (STAT) signaling inhibition (155). The cross-talk between ILCs and epithelial cells is also important to promote airway epithelial repair and lung tissue homeostasis following acute lung damage (156).

## Conclusions

Due to its permanent exposure to both innocuous foreign antigens and immunostimulatory molecules, the lung has developed mechanisms, which we called “CONTRA” mechanisms, to prevent unwanted immune responses towards these harmless molecules (**Figure 2**). Besides some lung immune cells, like IMs whose main function known so far is dedicated to immunoregulation, most of the lung cells display plasticity that enable them to exert regulatory function under certain conditions while promoting immune responses in others. Even though progress has been made in our understanding of mechanisms preventing the development of unnecessary immune responses, all of these regulatory mechanisms are probably far from being discovered. Indeed, cell types known as pro-inflammatory ones for decades (e.g. eosinophils) are more and more reported with regulatory functions. Future research should allow a better understanding of these immune brakes and could therefore be beneficial for the development of therapeutic strategies against lung diseases.

## Author contributions

FB and CS drafted the manuscript. CS drew the figures and wrote the manuscript under the supervision of FB. FB reviewed the manuscript. All authors contributed to the article and approved the submitted version.
